# Relevant pericardial effusion caused by cytomegalovirus infection in an immunocompetent patient: a case report

**DOI:** 10.1186/s13256-017-1542-6

**Published:** 2018-01-19

**Authors:** Tabea Hutter, Dirk Springe, Lukas Ebnöther, Marcos Delgado

**Affiliations:** Intensive care unit, Department of Anaesthesiology, Bürgerspital Solothurn, Schöngrünstrasse 42, CH-4500 Solothurn, Switzerland

**Keywords:** Cytomegalovirus, Immunocompetent, Severe pleural effusion

## Abstract

**Background:**

Cytomegalovirus infection is known to cause symptomatic disease in immunocompromised patients, while an infection in immunocompetent individuals normally causes few or no symptoms. We present the case of an immunocompetent adult patient with unexpected severe evolution.

**Case presentation:**

An otherwise healthy, 72-year-old Caucasian woman presented with complaints of progressive shoulder pain and dyspnoea on exertion. The blood test results showed elevated inflammation parameters and elevated hepatic transaminase levels. Radiologic examinations were carried out, and the computed tomography scan revealed a hepatomegaly and a chest X-ray showed evidence of a unilateral pleural effusion. A transthoracic echocardiography detected pericardial effusion with consecutive hemodynamic changes. Since it was considered that using ultrasound-guided pericardiocentesis could significantly increase the risk of liver injury due to hepatomegaly, a pericardial window was performed instead. Further investigation showed that our patient tested positive for an acute cytomegalovirus infection in the serologic tests. Laboratory findings included new evidence of immunoglobulin M seroconversion and high immunoglobulin G avidity, so we considered the possibility that a former cytomegalovirus infection may be coexisting with a new cytomegalovirus reinfection.

**Conclusions:**

In immunocompetent individuals, a symptomatic cytomegalovirus primary infection or reinfection should be considered in patients presenting with pericardial effusion and serositis.

## Background

Cytomegalovirus (CMV) infection in immunocompromised patients is known to cause symptomatic disease. In immunocompetent patients, a CMV infection usually proceeds without symptoms or provokes symptoms resembling infectious mononucleosis. However, several reports which consider a more severe clinical course of disease in otherwise healthy people have been published [1–3]. We report the case of a healthy immunocompetent woman who presented in our hospital with hemodynamically relevant pericardial effusion caused by an acute CMV reinfection showing new immunoglobulin M (IgM) seroconversion. This case emphasizes the fact that a CMV infection or reinfection, must be considered as a cause of a pericardial effusion in immunocompetent individuals.

## Case presentation

### Anamnesis

A 72-year-old Caucasian woman presented at her general practitioner complaining that she had been suffering from progressive shoulder pain for 2 weeks and dyspnea on exertion for 1 week. Our patient´s past medical history included a case of arterial hypertension, treated with cilazapril and atenolol/chlorthalidone, as well as dyslipidemia, treated with atorvastatin. Our patient has been smoking about 5 cigarettes each day for the last 30 years but she does not consume alcohol on a daily basis. Our patient is retired and lives with her husband. They have two children and three grandchildren. In the initial examination carried out by her general practitioner, a blood sample was taken and a chest X-ray was carried out, showing elevated inflammation parameters and a large unilateral pleural effusion. As our patient´s general condition deteriorated she was admitted to the hospital.

### Investigations

Upon admission to the hospital our patient was afebrile (with a temperature of 37.3 °C). Our patient´s blood pressure was 91/59 mmHg and she had a heart rate of 82 beats/min (given that she was undergoing treatment with atenolol). A 2/6 systolic heart murmur was evident from her clinical examination. There was no visible engorgement of the neck veins, nor signs of lower leg edema. Her breath sounds were attenuated on the left side and her neurologic examination was normal.

**Fig. 1:**
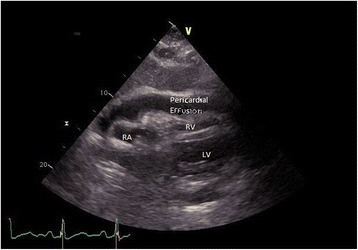
Transthoracic echocardiogram with subcostal view. Pericardial effusion with compression of the *RA* right atrium, *RV* right ventricle, *LV* left ventricle

**Fig. 2:**
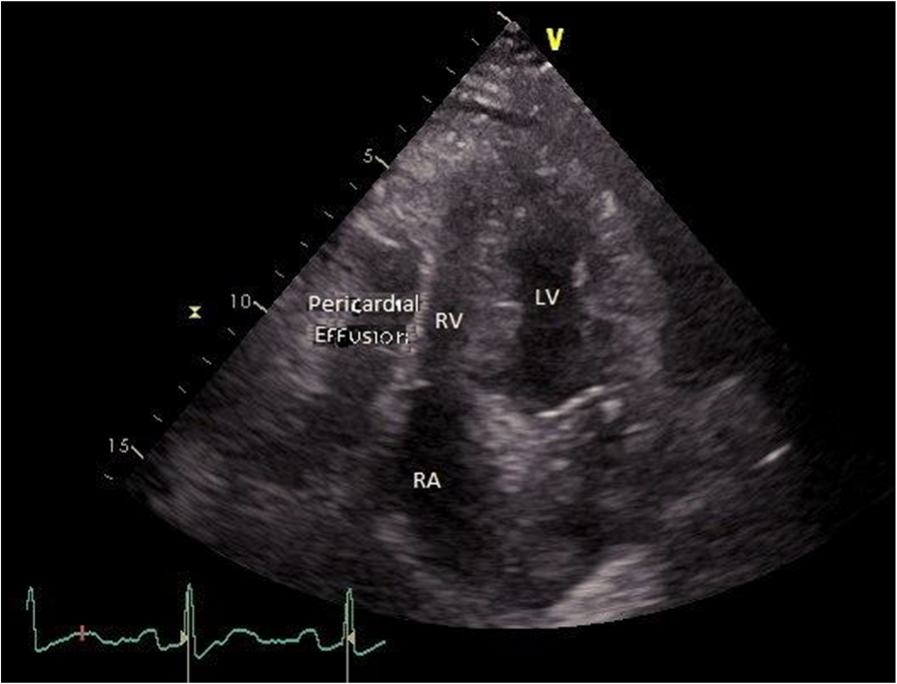
Apical four chamber transthoracic echocardiogram view. Displacement of the *RA* right atrium, *RV* right ventricle, *LV* left ventricle

Her blood results showed elevated C-reactive protein (CRP, upon admission 256.7 mg/L; reference < 7.5 mg/L) and leukocytosis (leukocytes upon admission 21.5 10^9/L, reference 3.5–10.0 10^9/l) with slight monocytosis present in the leukogram. Additionally, she showed elevated levels of aspartate amino transferase (ASAT) and alanine amino transferase (ALAT) as well as alkaline phosphatase and gamma-glutamyltransferase (GGT), whilst her bilirubin levels were within the normal range. She presented with slightly impaired renal function upon admission but this was normalized after hydration. The aspiration of the pleural effusion revealed exudate without signs of a bacterial infection (cell number 4 10^9/L, out of which 85.5% were polynuclear cells and 13.5% were mononuclear cells; pH 7.57, glucose 7.5 mmol/L, protein 39 g/L, lactate dehydrogenase [LDH] 213 U/L; no growth of microorganisms). A transthoracic echocardiography revealed pericardial effusion with consecutive hemodynamic changes but no pericardial tamponade. The left ventricle was normal in shape with normal systolic function (left ventricular ejection fraction 60%), but there was evidence of dysfunctional relaxation. The heart valves were normal. The right ventricle was normal in shape and function (Figs. 1 and 2).

A computed tomography (CT) scan showed dilatation of the inferior vena cava and of the liver veins, as well as a hepatomegaly, resulting either from venous congestion or from an inflammatory process.

Several additional blood tests were done in order to rule out a rheumatologic etiology: rheumatoid factor, anti-citrullinated protein antibody, anti-nuclear antibody, anti-neutrophil cytoplasmic antibody, double-stranded deoxyribonucleic acid (dsDNA), anti-Sm antibody, anti-mitochondrial antibody, and complements C3 and C4. The results of all of these tests were normal. To determine a possible infectious cause, serologic testing was performed for hepatitis B and C, human immunodeficiency virus (HIV), Borrelia burgdorferi, Epstein-Barr virus (EBV), CMV, chlamydia, enterovirus, and mycoplasma. The results showed an acute CMV reinfection.

### Evolution

As a result of the hemodynamic changes caused by the pericardial effusion, our patient was admitted to the intensive care unit (ICU) where she was stabilized with intravenous fluids and vasoactive medications. Our patient’s respiratory status remained stable with oxygen administered through a nasal cannula and she did not show any symptoms of any neurological disorders. Given that an ultrasound-guided pericardiocentesis carried a high risk of liver injury due to hepatomegaly, the surgical team performed a pericardial window. Pericardial and liver biopsies were obtained during the procedure. The histologic results showed mild hepatic steatosis and signs of chronic fibrinous pericarditis. The cytopathology report of the pericardial effusion showed reactive mesothelial changes and signs of a lymphohistiocytic reaction. Our patient’s post-interventional clinical outcome was uncomplicated, and she was transferred from the ICU to the ward. The results of the previously ordered rheumatologic tests were all negative. The serology results were positive for an acute CMV infection. The IgM seroconversion occurred during her hospital stay (negative result upon admission) while the immunoglobulin G (IgG) levels were elevated in all blood samples. The IgG avidity was high (CMV IgG avidity 0.462; reference < 0.25), confirming the coexistence of a past CMV infection with a new CMV reinfection due to IgM seroconversion.

As our patient was not known to suffer from immunodeficiency, no antiviral therapy was indicated. Our patient left the hospital, against medical advice, before a CMV polymerase chain reaction (PCR) or additional tests could be ordered.

After she left the hospital, her infection parameters and hepatic transaminase levels normalized completely, and there was no recurrence of the pleural or pericardial effusions. In a blood sample taken 19 months after hospitalization, her transaminase levels and infection parameters remained within the normal range, but a new CMV serology was not performed by the doctor in charge.

## Discussion

We report the case of an otherwise healthy, immunocompetent patient, who suffered severe complications from an acute CMV reinfection. In immunocompetent patients, a CMV infection usually provokes symptoms resembling an infectious mononucleosis or does not cause any symptoms at all. However, a few reports have been published about severe complications, as in our case.

Cytomegalovirus belongs to the herpes viruses’ family and has a linear, double-stranded DNA [4]. The overall prevalence of CMV is high. A study conducted in Germany from a population of healthy blood donors found a prevalence of 45.8%, with slightly more females than males being carriers of the virus [5]. Other sources suggest a prevalence of about 60%. In developing countries, the prevalence is predicted to be approximately 90% [4]. This infection is most commonly transmitted horizontally through saliva, sexual intercourse, blood transfusion or through an organ transplantation; nevertheless, vertical transmission is possible as well [4]. Immunocompromised patients are at an increased risk of suffering from symptomatic CMV disease.

The primary infection is followed by a latent phase of infection and the virus can be reactivated at a future point in time, particularly in the case of an impairment of the immune system [4]. A seroconversion of the IgM combined with a low IgG avidity and the corresponding clinical findings confirm a recent (within the previous 4 months) CMV primary infection. In contrast, a high IgG avidity suggests a CMV infection earlier on (more than 4 months ago). In the case of a CMV reinfection with a new virus strain, the laboratory findings include a new IgM seroconversion (which can take place up to 2 weeks after exposure to CMV) and high levels of IgG with a high IgG avidity. That was the constellation of symptoms seen in the patient discussed in this case report. A reinfection is not a rare event – a prospective study conducted on a population of healthy, seropositive women found a reinfection in 29% of the participants [6].

Immunocompetent patients with a primary CMV infection either do not suffer from any symptoms or experience symptoms resembling an infectious mononucleosis caused by the EBV. Tonsillitis and lymphadenopathy occur less frequently in patients with a CMV infection than in patients with EBV. Asthenia, arthralgia, and fever are common in patients with a CMV infection, and laboratory findings typically include lymphocytosis and atypical lymphocytes. A slight impairment of the liver function with a small elevation of the transaminases occurs frequently as well [1, 4]. However, in rare cases, severe complications can also occur in immunocompetent patients. Myopericarditis, pericardial effusion, hepatitis [1, 2], colitis, and central nervous system involvement with encephalitis, meningitis or transverse myelitis are possible. In rare cases, pulmonary or ocular involvements have been described. Furthermore, different hematologic disorders including symptomatic thrombocytopenia, hemolytic anemia, disseminated intravascular coagulation, myelodysplastic changes, pancytopenia, and ruptured spleen can occur [3].

The appropriate therapy for a hemodynamically relevant pericardial effusion consists of an immediately performed, ultrasound-guided pericardial puncture and drainage of the effusion in the ICU [7, 8]. In our patient, surgical intervention was preferred due to the existing hepatomegaly and increased risk of a liver injury.

As an acute CMV infection usually shows a self-limiting, oligosymptomatic course in immunocompetent individuals, antiviral therapy is typically not indicated for this group. Several case studies reported the successful use of antiviral medications such as ganciclovir, valganciclovir and foscarnet in previously healthy patients with severe organ-specific complications or a prolonged disease course. At present, there are no recommendations concerning the dosage and duration of antiviral therapy in immunocompetent patients. The severity of the disease has to be balanced against the medical toxicity separately for each individual [3]. In our patient, the clinical course was uncomplicated, and no antiviral therapy was required.

## Conclusions

In immunocompetent individuals, a symptomatic CMV primary infection or reinfection should be considered as part of the differential diagnosis when patients present with pericardial effusion and serositis. Symptomatic therapy is indicated for immunocompetent patients with acute CMV disease. In the literature, recommendations concerning an antiviral therapy regimen are still being discussed.

## Abbreviations

ALAT: Alanine aminotransferase
ASAT: Aspartate aminotransferase
CMV: Cytomegalovirus
CRP: C-reactive protein
EBV: Epstein-Barr virus
GGT: Gamma-glutamyltransferase
HIV: Human immunodeficiency virus
ICU: Intensive care unit
Ig: immunoglobulin
PCR: Polymerase chain reaction
